# Early-life painful and stressful exposures and neurodevelopment in preterm infants

**DOI:** 10.3389/fped.2026.1820878

**Published:** 2026-05-13

**Authors:** Tingting Zhao, Aolan Li, Ming-Hui Chen, Adam Matson, Xiaomei Cong

**Affiliations:** 1 School of Nursing, Columbia University, New York, NY, United States; 2 School of Nursing, Yale University, Orange, CT, United States; 3 Department of Statistics, University of Connecticut, Storrs, CT, United States; 4 Division of Neonatology, Connecticut Children’s Medical Center, Hartford, CT, United States; 5 Department of Pediatrics, University of Connecticut School of Medicine, Farmington, CT, United States

**Keywords:** composite pain scores, mother's own milk, neurodevelopment, painful and stressful exposures, preterm

## Abstract

**Introduction:**

Early-life painful and stressful exposures have been associated with neurodevelopmental outcomes in preterm infants, however, findings regarding the roles of race, sex, and mother's own milk (MOM) intake in these associations are inconsistent.

**Method:**

A cohort of 196 infants (28 to 32 weeks' gestation) was followed from birth to 2 years corrected age (CA) and included in the study analyses. Painful and stressful exposures during the first 28 days of life were quantified using composite pain scores derived from the Neonatal Infant Stressor Scale. Neurodevelopment was assessed using the NICU Network Neurobehavioral Scale at 36–38 weeks postmenstrual age and the Bayley-III and BITSEA at 1- and 2- years CA. Linear mixed-effects and regression models were applied.

**Results:**

Higher painful exposures during the first 28 days were associated with poorer self-regulation, attention, greater stress and lethargy (*p* < 0.05) at 36 to 38 weeks PMA. Among Black infants, higher composite pain scores predicted lower language, motor scores at 1- and 2- years CA and lower cognitive scores at 1 year CA (*p* < 0.05). Male infants with higher composite pain scores had lower motor scores at 1 year CA compared to female infants (*p* < 0.05). Greater MOM intake was linked to lower composite pain scores (*p* < 0.001) but did not moderate pain–neurodevelopment association.

**Conclusion:**

Black and male infants experienced higher painful exposures compared with White and female infants. Greater MOM intake showed a trend toward being protective, suggesting a potential role in individualized neonatal care.

## Introduction

Preterm infants [≤37 weeks gestational age (GA)], especially those born very preterm (≤32 weeks GA), often require prolonged hospitalization in the neonatal intensive care unit (NICU) (19). During this period, they can be exposed to numerous life-saving but painful and stressful procedures, including heel sticks (14), diapering (6), repositioning (4), Nil per os (NPO) (13), intravenous insertions, and frequent handling (34). Since the first 28 days of life represent a critical window of brain plasticity in preterm infants (31), studying the association between early-life painful exposures and neurodevelopment could provide important insights into susceptibility factors that modify the adverse effects of early-life stress.

Evidence shows that early-life painful exposures can influence brain development at both structural and functional levels (7, 10, 26). Structurally, repeated exposure to early-life pain has been associated with reduced thalamic volume, particularly in the somatosensory thalamus, between 32 and 40 weeks postmenstrual age (PMA) (10), as well as decreased amygdala and thalamus volumes by 8 years of age (7). Functionally, such exposures have been linked to altered brain connectivity, including reduced global efficiency, local efficiency, and regional connection strength, as assessed by magnetic resonance imaging (26). In addition, stress-related symptoms, such as jitteriness and exaggerated startle responses observed at 36 to 38 weeks PMA, and delays in cognitive, motor, and language development at 1 and 2 years of corrected age (CA) also have been associated with early-life painful and stressful exposures (34).

These associations may vary by infant race, sex, and factors such as mother's own milk (MOM) intake (35). Black infants, in particular, are disproportionately exposed to early-life adversities, including environmental stressors (e.g., maternal smoking), adverse socioeconomic status, nutritional deficiencies, and other NICU-related stressors that may impair neurodevelopment (2, 9, 24, 25). However, differences in responses to painful and stressful exposures among racial groups and their impact on neurodevelopmental outcomes remain poorly understood. Although animal study suggested sex-differentiated responses or recovery to early life painful exposures (20), sex-based differences in neurodevelopmental vulnerability to early-life stress among preterm infants have not been well characterized. Another important and understudied modifier of exposure to early-life stress is MOM intake. Our previous findings suggest that higher MOM (70% or more) intake may buffer the negative effects of painful and stressful exposures on neurobehavioral outcomes in the NICU (35). However, its potential role in shaping long-term neurodevelopmental trajectories in preterm infants impacted by exposure to early-life pain remains underexplored.

This study aims to investigate the associations of early-life painful and stressful exposures and neurobehavioral responses during the NICU stay, as well as neurodevelopmental outcomes through 2 years CA in preterm infants. We also assess whether these associations are influenced by race, sex, and the MOM intake. By examining these associations and modulations, our research seeks to deepen understanding of disparities in early-life adversity and inform targeted strategies to promote infant health outcomes.

## Method

### Study design and participants

We conducted a cohort study to examine the associations between early-life painful and stressful exposures and neurodevelopment in preterm infants during their NICU stay and at 1 and 2 years of CA. Infant race, sex, and the MOM intake were adjusted. Institutional Review Board approval was obtained. Between September 2017 to July 2022, a total of 215 medically stable preterm infants were recruited from two Level III and IV NICU sites in the Northeastern U.S. Inclusion criteria: Infants were 1) born at 28 to 32 weeks GA 2) consented by parents (≥ 18 years). Exclusion criteria: infants had 1) known congenital or chromosomal abnormalities; 2) severe periventricular/intraventricular hemorrhage (grade III or higher); 3) undergone surgery; and/or 4) prenatal exposure to illicit substances.

### Demographic and medical characteristics

Infant demographic data, including race, sex, ethnicity, birth GA, birth weight, length, head circumference, were collected from Electronic Medical Record (EMR). Medical data, including antibiotic use (before and after the first 3 days of life), delivery method, history of preterm premature rupture of membrane (PPROM), and the Score for Neonatal Acute Physiology with Perinatal Extension II (SNAPPE-II) were also collected from EMR.

### Mother's own milk intake

Research nurses recorded daily MOM frequency and volume across three shifts. For each infant, the total MOM intake volume over the first 28 NICU days was averaged to represent daily MOM intake.

### Neonatal infant stressor scale (NISS)

Early-life painful and stressful exposures were documented by research nurses using the Neonatal Infant Stressor Scale (NISS) during the first 28 NICU days (8, 23). The NISS categorizes exposures as acute (e.g., diaper changes, heel sticks) or chronic (e.g., intranasal oxygen, intravenous catheter indwelling). Acute exposures were documented by frequency and chronic events by durations (in hours). The severity of each event (rated from 2, “a little” to 5 “extreme”) was determined using the validated NISS. The ratings reflect the intensity of the painful or stressful exposures, as assessed by expert NICU clinicians based on the original NISS criteria (23). Average daily unweighted NISS scores were calculated as the sum of daily frequencies or durations divided by 28 days. Weighted NISS scores multiplied each event's frequency (acute) or duration (chronic) by severity, with chronic duration further multiplied by 8 h per shift over the 28-day period to derive composite pain scores. The composite pain scores were then divided by 28 to obtain the daily average weighted NISS scores over 28 days.

### NICU neonatal neurobehavioral scale (NNNS)

Certified research nurses administered the NICU Neonatal Neurobehavioral Scale (NNNS) (17) at 36 to 38 weeks of PMA when infants were medically stable. The NNNS consists of 115 items yielding 13 summary scores assessing neurological integrity and behavioral function across three main domains (e.g., neurological items for tone and reflexes; behavioral items for state, sensory, and interactive processes; and stress/abstinence related items). Our analyses focused on four scores: STRESS/ABSTINENCE (higher = more stress signs), LETHARGY (lower = more lethargy reactivity), ATTENTION (higher = better tracking auditory and visual stimuli), and SELF-REGULATION (higher = better regulation of physiological, motor, and attentional states).

### Bayley scales of infant and toddler development, Third Edition (Bayley-III)

The Bayley Scales of Infant and Toddler Developmental, Third Edition (Bayley-III) (3), was administered by neonatologists at 1 and 2 years of CA to assess Cognitive, Language, and Motor development. Each domain generates a standardized score, with higher scores indicating better outcomes.

### Brief infant toddler social emotional assessment (BITSEA)

The BITSEA assesses early psychosocial challenges in infants and toddlers via two subscales: the Problem Scale (31 items) and the Competence Scale (11 items) (16). At the two-year CA follow-up, parents completed the BITSEA and item responses were summed for each scale. Problem scores range 0 to 62, and the Competence scores range from 0 to 22. Higher scores on the Problem Scale and lower scores on the Competence Scale reflect less favorable developmental outcomes.

### Statistical analysis

We examine associations between early life painful and stressful exposures (NISS), and neurobehavioral outcomes (NNNS) at NICU and neurodevelopmental outcomes (Bayley III and BITSEA) at 2 years CA across. The composite pain scores summed weighted acute (level 3 to 5) and chronic (level 2 to 5) pain scores. Linear mixed-effects models assessed the relationship between Bayley III scores and composite pain scores, adjusting for sex, race, ethnicity, MOM amount, mode of delivery, birth GA, SNAPPE-II score, and PPROM status, with a random intercept included to account for within-subject correlations. An overall model was fitted without interaction terms, and separate sub-models were subsequently developed to individually examine interactions between visit time and race, sex, MOM amount, and the composite pain scores. A Box-Cox transformation (*λ* = 2) was applied to the Bayley language and cognitive scores to improve model assumptions and robustness (11). BITSEA Competence and Problem scores were dichotomized by comparing each infant's score to the established cutoff: scores were classified as 1 if the ratio exceeded 1 (indicating concern) and 0 otherwise. These binary outcomes were analyzed using binomial logistic regression models with same adjustments. Linear regression models were used to examine associations between NNNS subscales and composite pain scores, also including an interaction between race, sex, MOM amount, and composite pain scores. All statistical analyses were performed using R (version 4.5.1), with significance defined as *p* < 0.05.

## Results

### Demographic and medical characteristics

A total of 196 preterm infants who were identified as Black or White were included in the analysis (Table 1). Among them, 59.2% were male, 75.0% were non-Hispanic, and 26.0% identified as Black. Cesarean delivery occurred in 69.9% of cases, and 23.0% of infants were born following preterm premature rupture of membranes (PPROM). The average birth weight was 1,075.7 grams (SD = 346.6), with an average body length of 36.5 cm (SD = 4.0) and head circumference of 25.4 cm (SD = 2.6). The mean SNAPPE-II score was 23.7 (SD = 17.5). Additionally, 94.9% of infants received antibiotics within the first three days of life, and 48.5% received antibiotics during the last three days of the observation period. Compared to White infants, Black infants were significantly more premature, had lower birth weights, shorter birth length, smaller head circumferences, and higher SNAPPE-II scores, all with *p*-values <0.001 (Table 2).

**Table 1:** Infant demographic characteristics (*N* = 196).

Variables	Total (*N* = 196)
Sex	
Female	80 (40.8%)
Male	116 (59.2%)
Ethnicity	
Hispanic	49 (25.0%)
Non-Hispanic	147 (75.0%)
Delivery	
C-section	137 (69.9%)
Vaginal	59 (30.1%)
PPROM	
Yes	45 (23.0%)
Antibiotic used first 3 days	
Yes	186 (94.9%)
Antibiotic used after 3 days	
Yes	95 (48.5%)
	Mean (SD)
Birth GA (week)	28.2 ± 2.5
Birth weight (g)	1,075.7 ± 346.6
Birth body length (cm)	36.5 ± 4.0
Birth HC (cm)	25.4 ± 2.6
SNAPPEII	23.7 ± 17.5

PPROM, Preterm premature rupture of membranes; HC, head circumference; GA, gestational age; SNAPEII, Score for Neonatal Acute Physiology with Perinatal Extension-II.

**Table 2:** Infant demographic characteristics by race (*N* = 196).

Variables	Black (*n* = 51)	White (*n* = 145)	*p*-value
Delivery			
C-section	40 (78.4)	97 (66.9)	0.172
Vaginal	11 (21.6)	48 (33.1)	
PPROM			
Yes	8 (15.7)	37 (25.5)	0.214
No	43 (84.3)	108 (74.5)	
Antibiotic used first 3 days			
Yes	49 (96.1)	137 (94.5)	0.940
No	2 (3.9)	8 (5.5)	
Antibiotic used after 3 days			
Yes	9 (17.6)	19 (13.1)	0.572
No	42 (82.4)	126 (86.9)	

	Mean (SD)	Mean (SD)	*p*-value
Birth GA (week)	27.2 (2.4)	28.5 (2.4)	0.001
Birth weight (g)	902.8 (285.1)	1,136.5 (346.6)	<0.001
Birth body length (cm)	34.8 (3.6)	37.1 (3.9)	<0.001
Birth HC (cm)	24.1 (2.4)	25.9 (2.6)	<0.001
SNAPEII	31.9 (19.4)	20.9 (15.8)	<0.001

PPROM, Preterm premature rupture of membranes; HC, head circumference; GA, gestational age; SNAPEII, Score for Neonatal Acute Physiology with Perinatal Extension-II.

## Painful and stressful exposures

**Figure 1:**
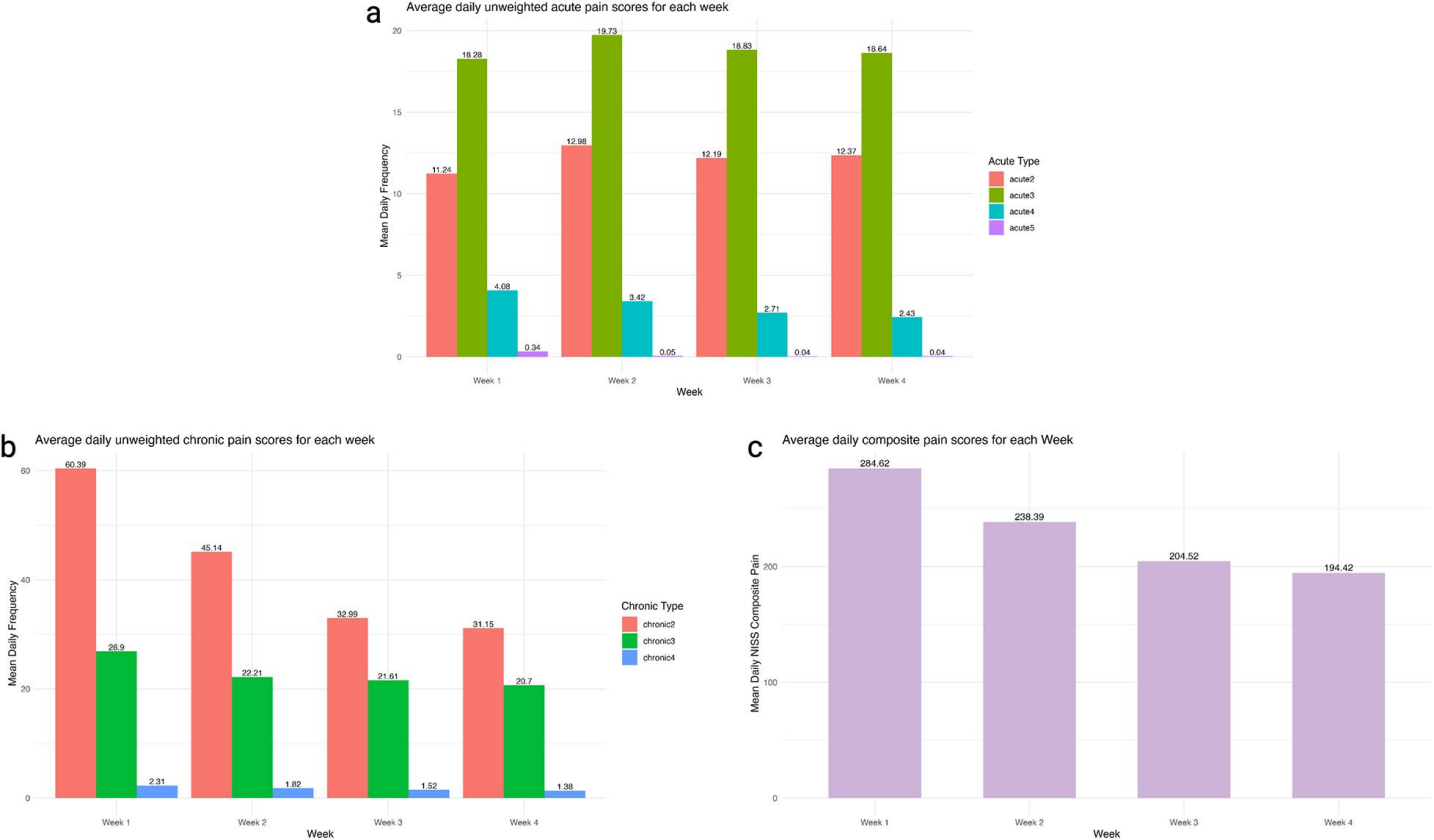
**(a)** average daily unweighted acute pain **(a)**, chronic pain **(b)**, and composite pain scores **(c)** in first 4 weeks after birth.

During the first 28-day, infants experienced an average of 964.6 acute painful and stressful exposures (SD = 214.8), corresponding to 34.5 events per day per infant (SD = 7.6) (Supplementary Table S1). Level 3 events accounted for 32.6% (SD = 11.4%) of all events, averaging 19.0 per day per infant (SD = 3.7) (Supplementary Table S1). Acute Level 2 events increased slightly from week 2 through week 4, while Level 3 events increased modestly in week 2, then decreased but remained above week 1 levels. In contrast, Level 4 events were highest in week 1 and declined steadily through week 4 (Figure 1a).

Infants experienced 1,893.4 h (SD = 693.0) of chronic painful and stressful exposures over 28 days, averaging 67.8 h per day (SD = 24.8) (Supplementary Table S1). The majority of chronic painful and stressful exposures were rated at Level 2 (67.8%, SD = 11.8%) and Level 3 (32.6%, SD = 11.4%) (Supplementary Table S1), totaling 65.8 h per day per infant (SD = 21.5) (Supplementary Table S1). Chronic painful and stressful exposures at all levels gradually declined over four weeks, with the most noticeable reduction observed at Level 2 (Figure 1b). Overall, combined acute and chronic painful and stressful exposures were highest in week 1 and steadily declined through week 4 (Figure 1c).

### Composite pain scores by race, sex, and MOM intake

**Figure 2:**
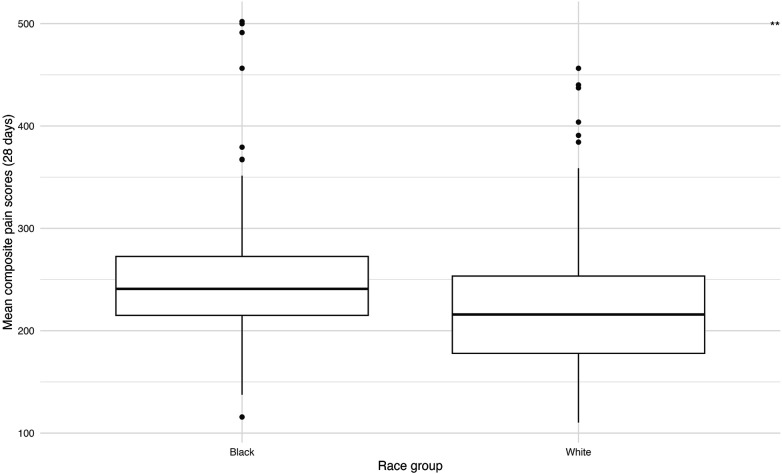
Mean composite pain scores within first 28 days after birth between different Black and White infants.

**Figure 3:**
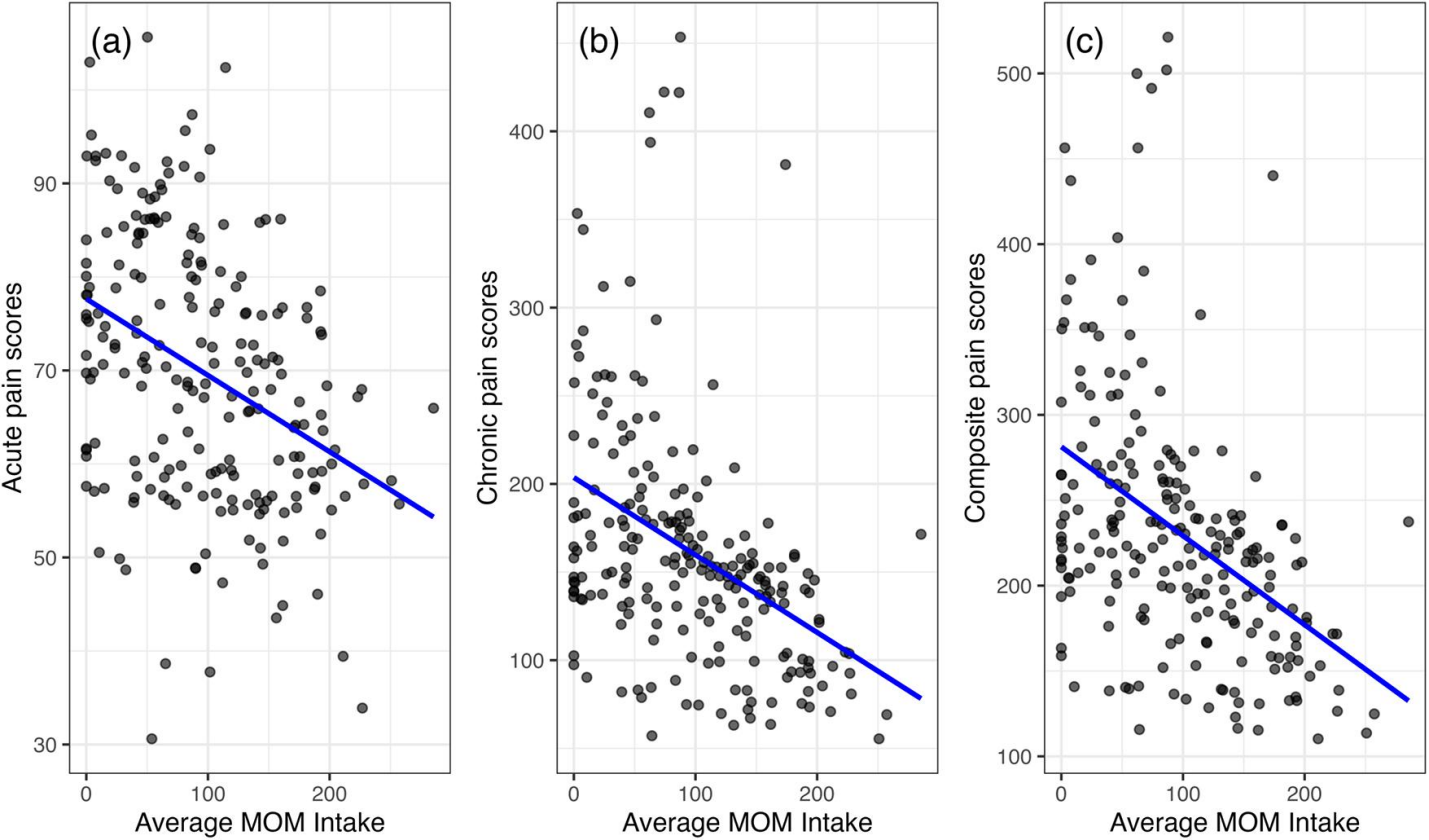
The associations between acute pain exposure **(a)**, chronic pain exposure **(b)**, and composite pain score **(c)** and average mother's own milk (MOM) intake.

Black infants had significantly higher average unweighted and weighted daily acute (*p* = 0.028, 0.002) and chronic (*p* = 0.011, 0.013) painful and stressful exposures, as well as higher composite pain scores (*p* = 0.006) compared to White infants (Figure 2, Supplementary Table S2). Males and females had similar total unweighted acute (981.0 vs. 940.7) and chronic (1,879.4 vs. 1,913.7) exposures, with no significant differences (Supplementary Table S3). Higher average MOM intake during the first 28 days was associated with lower acute (*r* = –0.38), chronic (*r* = –0.44) pain scores, and composite pain scores (*r* = –0.46), all *p* < 0.001 (Figure 3).

### Composite pain scores and neurobehavioral and neurodevelopmental outcomes

Higher composite pain scores were significantly associated with increased stress (*p* = 0.006) and lethargy (*p* < 0.001), and lower self-regulation (*p* = 0.007) and attention (*p* = 0.012) (NNNS subscales). However, no correlations were observed between composite pain scores and either Bayley-III or BITSEA scores.

### Composite pain scores and neurobehavioral and neurodevelopmental outcomes by race

Among Black infants, higher composite pain scores remained significantly associated with increased stress (*p* = 0.015) and lethargy (*p* < 0.001) and decreased attention (*p* = 0.010) and self-regulation (*p* = 0.031). They were also significantly associated with poorer Language, Motor, and Cognitive development (Bayley-III) at 1-year CA (*p* = 0.014, 0.009 & 0.018), and poor Language and Motor development (Bayley-III) at 2-year CA (*p* < 0.001 & *p* = 0.025). Among White infants, significant associations were observed only for arousal (*β* = −0.004, *p* = 0.004) and lethargy (*p* = 0.011). No associations were found with BITSEA Competence or Problem scores at 2 years CA.

### Composite pain scores and neurobehavioral and neurodevelopmental outcomes by Sex

Higher composite pain scores were associated with increased stress (*p* = 0.022 & 0.044) and lethargy (*p* = 0.006 & 0.010) in both male and female infants, with females also showing decreased attention (*p* = 0.016) and males showing decreased self-regulation (*p* = 0.027) and poorer motor outcomes (*p* = 0.039) at 1 year CA. No associations were found between composite pain scores and BITSEA scores at 2 years CA in male or female infants.

### Composite pain scores and neurobehavioral and neurodevelopmental outcomes by MOM intake

Interactions between composite pain scores and average MOM intake were not significant for any neurodevelopmental outcomes. A marginal trend was observed for language development at 1 years CA (*p* = 0.094), suggesting a possible association between higher MOM intake and reduced impact of composite pain scores, though these findings should be interpreted cautiously.

## Discussion

Our study identified that Black infants with higher composite pain scores exhibited poorer neurodevelopment during the NICU stay and delayed motor and language development at 1- and 2-year follow-ups. These disparities may partly reflect differences in birth health status, such as younger GA and smaller anthropometric measurements (1), as well as caregiver-related factors, including communication challenges and language barriers (12). Importantly, broader maternal and socioeconomic factors, such as differences in income, education, insurance type, and neighborhood resources, can influence access to prenatal care, nutrition and resources that support infant neurodevelopment (2, 32, 36). Because these factors were not measured in our study, observed disparities between Black and White infants may reflect both biological and environmental influences, rather than race alone, and should be interpreted with caution.

Preterm infants are at higher risk to early life painful and stressful exposures, especially during the first 28 days of NICU stay, when they often undergo frequent invasive procedures such as intubation and heel sticks (28, 34) As infants become clinically stable, prolonged medical procedures become more common, with the focus of care shifts toward sustained supportive procesures (21). Although these procedures may cause less immediate pain, their continuous presence can lead to cumulative, low-level stress, contributing to the overall burden of chronic painful and stressful exposures in the NICU environment (21).

Our study revealed race and sex disparities in painful and stressful exposures, Black preterm infants experienced significantly higher composite pain scores than White infants, reflecting greater exposure to early life adversity. This may be partly due to their younger GA and smaller anthropometrics at birth, which increase medical procedures needs. Additionally, although both sexes showed early neurobehavioral vulnerability at 36 to 38 PMA, male infants demonstrated greater long-term neurodevelopmental risk (20, 29). Despite larger birth weight, body length, and head circumference, males demonstrated a slower growth trajectory in early life (34, 35). This disadvantage was associated with a lower proportion of MOM received by males during the first 9 weeks of life (35). The observed slower catch-up growth and decreased MOM intake (35) may be insufficient to buffer the negative effects of early painful and stressful exposures in male infants.

Interestingly, among the various types of painful and stressful exposures, diaper changes and repositioning, both classified as level 3 (moderate pain), occurred most frequently. Although diapering is routine NICU nursing care, it can disrupt infants' sleep-wake patterns, elevate heart and respiratory rates, and lower body temperature, indicating physiological stress (6). These effects may stem from infants' limited body fat, which compromises their ability to regulate body temperature during diaper changes and can lead to a significant heat loss (30). Sleep disruption may further heighten stress and heart rate. Thus, some studies recommend minimizing unnecessary diaper changes to reduce caregiving-related stress (30). Similarly, repositioning is standard NICU practice including supine, prone, lateral, and semi-reclined positioning (4). For infants receiving continuous positive airway pressure (CPAP), the supine position supports respiratory function and reduces the risk of sudden infant death syndrome (SIDS) (4). However, it may also be the most stressful position for infants (22). These conflicting findings underscore the need for individualized positioning strategies tailored to each infant's clinical needs.

In our study, heel sticks (level 4, very painful) remained among the most frequent painful exposures in the NICU. Consistent with this, previous studies have shown that younger and smaller preterm infants may exhibit reduced responses to heel sticks, possibly due to increased vulnerability to repeated and prolonged chronic painful exposures, while their recover from painful stimuli is slower compared with older preterm infants (27). Furthermore, early exposure to heel sticks has been associated with adverse physiological and behavioral responses, for example, infants exposed to heel sticks in the first few days of life demonstrated significantly lower SpO_2_ levels and longer crying durations (14).

Following early-life adversities and repeated painful and stressful exposures, some infants may be at risk for both short-term neurobehavioral disorders as well as long-term neurodevelopmental impairments (15, 34). In our study, higher composite pain scores were significantly associated with poorer neurobehavioral outcomes during the NICU stay, but this association was not observed at 1- and 2- year CA. This discrepancy may reflect that NNNS subscale scores are more sensitive to acute painful and stressful exposure in the neonatal period (33), whereas Bayley subscales capture long term development shaped by environment, caregiving, and broader social determinants of health, potentially attenuating early life effects (2, 5, 18).

## Strength and limitations

Our findings emphasize the urgent need to address both clinical practices and the systemic racial and sex disparities shaping care for preterm infants. Given the elevated risk of mortality and long-term developmental challenges among Black and male infants, equity-focused interventions are essential. Future research should integrate incorporate maternal social factors such as income, education, and healthcare access, to more comprehensively address these disparities. Clinicians and healthcare systems must remain vigilant to potential biases in care delivery and ensure that all infants receive equitable, compassionate, and developmentally supportive care. Our study did not examine maternal factors (e.g., socioeconomic factors, access to care, chronic stress) or key physiological indicators (e.g., heart rate, respiratory rate, body temperature, and arterial oxygen saturation) that may influence early-life adversities, painful and stressful exposures, and long-term neurodevelopmental outcomes in preterm infants. The smaller proportion of Black infants included in our study also limits generalizability. Future studies should consider maternal factors and key physiological indicators, including maternal milk by lactation stage and infant measures such as heart rate variability and cortisol, while recruiting larger and more racially balanced samples to strengthen statistical power and enhance applicability to preterm infant neurodevelopment.

## Conclusion and relevance

Our study underscores the significant impact of early life adversities, including painful and stressful exposures, on neurodevelopmental outcomes during NICU hospitalization. Among Black infants, these exposures were associated with poorer motor skills, delayed language development, and difficulties with emotional regulation that persist into toddlerhood. We also identified racial disparities in the frequency of certain painful and stressful procedures, such as diapering, position change and nil per os (NPO), with Black infants experiencing these stressors more frequently than White infants. Male infants showed particular vulnerability, with painful and stressful exposures linked to poorer motor development. Although MOM intake may have a modest buffering effect on the impact of painful and stressful exposures, the data do not support a clear protective effect, and adverse neurodevelopmental outcomes were still observed.

## Data Availability

The raw data supporting the conclusions of this article will be made available by the authors, without undue reservation.
