# Supplementary material for: Early-life painful and stressful exposures and neurodevelopment in preterm infants
Source: Front Pediatr. 2026 May 13;14:1820878. doi: 10.3389/fped.2026.1820878 (PMC13212501; doi:10.3389/fped.2026.1820878)
Supplement: Supplementary file 1 [file Table1.docx]

| **Domain** | **Measure** | **Mean ± SD** |
| --- | --- | --- |
| Acute | Total frequency (28 days) | 964.6 ± 214.8 |
| Acute | Daily frequency | 34.5 ± 7.6 |
| Acute | Level 3 proportion (%) | 32.6% ± 11.4 |
| Acute | Daily Level 3 frequency | 19.0 ± 3.7 |
| Chronic | Total duration (hours, 28 days) | 1893.4 ± 693.0 |
| Chronic | Daily duration (hours) | 67.8 ± 24.8 |
| Chronic | Level 2 proportion (%) | 65.5% ± 11.8 |
| Chronic | Level 3 proportion (%) | 32.6% ± 11.4 |
| Chronic | Daily Level 2+3 duration (hours) | 65.8 ± 21.5 |

**Supplementary Table 1.** Painful and stressful exposures during the first 28 days.
