# Supplementary material for: Early-life painful and stressful exposures and neurodevelopment in preterm infants
Source: Front Pediatr. 2026 May 13;14:1820878. doi: 10.3389/fped.2026.1820878 (PMC13212501; doi:10.3389/fped.2026.1820878)
Supplement: Supplementary file 2 [file Table2.docx]

| **Domain** | **Measure** | **Black (N=51)** | **White (N=145)** | **p** |
| --- | --- | --- | --- | --- |
| Acute | Events (28 days) | 1021.5 ± 236.4 | 944.6 ± 203.8 | 0.028 |
| Acute345 | Weighted | 75.6 ± 14.4 | 68.4 ± 13.7 | 0.002 |
| Chronic | Events (28 days) | 2133.3 ± 802.1 | 1809.0 ± 632.0 | 0.011 |
| Chronic | Weighted | 186.0 ± 79.1 | 154.8 ± 62.1 | 0.013 |
| Composite | Composite pain (weighted) | 261.6 ± 87.1 | 223.2 ± 69.7 | 0.006 |

**Supplementary Table 2.** Painful and stressful exposures by race.
