# Supplementary material for: Early-life painful and stressful exposures and neurodevelopment in preterm infants
Source: Front Pediatr. 2026 May 13;14:1820878. doi: 10.3389/fped.2026.1820878 (PMC13212501; doi:10.3389/fped.2026.1820878)
Supplement: Supplementary file 3 [file Table3.docx]

| **Domain** | **Measure** | **Female (N=80)** | **Male (N=116)** | **p** |
| --- | --- | --- | --- | --- |
| Acute | Events (28 days) | 940.7 ± 211.1 | 981.0 ± 216.7 | 0.198 |
| Acute345 | Weighted | 68.7 ± 13.4 | 71.3 ± 14.6 | 0.198 |
| Chronic | Events (28 days) | 1913.7 ± 682.7 | 1879.4 ± 702.7 | 0.735 |
| Chronic | Weighted | 165.3 ± 68.6 | 161.3 ± 68.1 | 0.681 |
| Composite | Composite pain (weighted) | 234.0 ± 74.8 | 232.6 ± 77.6 | 0.898 |

**Supplementary Table 3.** Painful and stressful events exposure by sex.
